# The “Centrality of Sepsis”: A Review on Incidence, Mortality, and Cost of Care

**DOI:** 10.3390/healthcare6030090

**Published:** 2018-07-30

**Authors:** Jihane Hajj, Natalie Blaine, Jola Salavaci, Douglas Jacoby

**Affiliations:** 1 Department of Nursing, Widener University, One University Pl, Chester, PA 19013, USA; 2 Department of Pharmacy, Penn Presbyterian Medical Center, 51 N 39th St, Philadelphia, PA 19104, USA; Natalie.blaine@uphs.upenn.edu (N.B.); jola.salavaci@uphs.upenn.edu (J.S.); 3 Department of Cardiology, Penn Presbyterian Medical Center, 51 N 39th St, Philadelphia, PA 19104, USA; Douglas.jacoby@uphs.upenn.edu

**Keywords:** severe sepsis, incidence, mortality, cost, postsepsis syndrome

## Abstract

Sepsis is a serious and fatal medical condition that has overburdened the US healthcare system. The purpose of this paper is to provide a review of published literature on severe sepsis with a distinct focus on incidence, mortality, cost of hospital care, and postdischarge care. A review of the nature of postsepsis syndrome and its impact on septic patients is also included. The literature review was conducted utilizing the PubMed database, identifying 34 studies for inclusion. From the evaluation of these studies, it was determined that the incidence of sepsis continues to be on the rise according to three decades of epidemiological data. Readmissions, mortality, and length of stay were all higher among septic patients when compared to patients treated for other conditions. The cost of treating sepsis is remarkably high and exceeds the cost of treating patients with congestive heart failure and acute myocardial infarction. The overall cost of sepsis is reflective of not only the cost of initial hospitalization but also the postdischarge care costs, including postsepsis syndrome and cognitive and functional disabilities that require a significant amount of healthcare resources long term. Sepsis and its impact on patients and the US healthcare system is a current quality-of-life and cost-burden issue that needs to be addressed with a greater focus on preventative strategies.

## 1. Introduction

Sepsis is a serious medical condition that has historically overburdened the US healthcare system. It is important to note the evolution of definitions used for sepsis in the latest guidelines. Sepsis, formerly “severe sepsis”, is defined as life-threatening organ dysfunction caused by a dysregulation of the host response following infection. Septic shock is a subset of sepsis which includes circulatory and metabolic dysfunction associated with higher mortality risk [1]. Hereinafter, “sepsis” and “severe sepsis” are used interchangeably, as the literature evaluated includes both terms. The burden of sepsis has been reported worldwide [1,2,3,4,5,6]. According to the most recent Center for Disease Control (CDC) report, it is estimated that sepsis affects around 1.5 million individuals in the United States annually, causing the death of 250,000 individuals and being responsible for 1 out of every 3 hospital deaths [7]. The treatment of sepsis can include fluid resuscitation, antimicrobial therapy, source control interventions, vasoactive medications, corticosteroids, blood products, and mechanical ventilation when necessary. The cost of each individual case of sepsis varies based on the presence or absence of septic shock as well as patient comorbidities and other patient-specific considerations. Sepsis is generally remarkably expensive to treat and has been associated with high readmission rates. Despite its overwhelming severity, cost, and mortality, sepsis has not significantly attracted the public’s attention. 

Since its inception in 2002, the Surviving Sepsis Campaign (SSC) has achieved several milestones. These include establishing standards of care through the development of treatment guidelines, increasing sepsis awareness, and improving diagnosis, treatment, and post-intensive care unit (ICU) care. Despite these advances, sepsis care remains quite expensive. While significant savings could be achieved, this has not been attained due to the economic dynamic of healthcare reimbursement that does not currently reward avoided costs but rather the potential for new revenues. For example, Medicare has reimbursed around $40,000 per case of severe sepsis that required mechanical ventilation compared to $12,000 for nonmechanically ventilated severe sepsis cases [8]. The objective of this paper is to provide a review of the literature that has addressed sepsis in regards to incidence and mortality, with a distinct focus on cost of care during and post hospitalization, as well as to provide a review of postsepsis syndrome and its burden on septic patients. 

## 2. Materials and Methods

**Figure 1:**
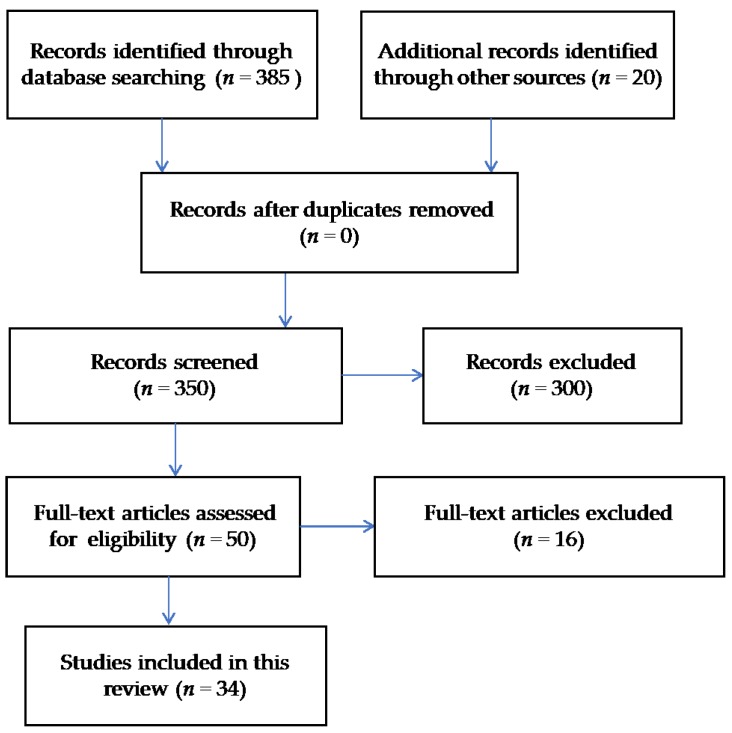
Literature search using the Preferred Reporting Items for systematic Reviews and Meta Analyses (PRISMA) flow diagram approach.

This was a comprehensive review of the literature that aimed to evaluate severe sepsis in the United States and internationally. The databases utilized for the purpose of this review were PubMed and Google Scholar. Web pages pertaining to the CDC and sepsis organizations were also examined for the purpose of this review. A wide and comprehensive list of subject headings were utilized, and these were: (1) severe sepsis; (2) incidence; (3) mortality; (4) cost; (5) readmissions; and (6) postsepsis syndrome. The search of manuscripts was restricted to original studies published in English and conducted in the United States and internationally which addressed the main topics pertaining to the purpose of this review. A detailed description of the literature search approach is summarized in Figure 1. A total of 34 studies were utilized for the purpose of this review. While a statistical assay was not used by these authors, the statistical findings of those studies reviewed are summarized and reported within the results section as a reference to assays performed externally.

The results of the literature review led to the emergence of various themes that highlighted the nature of severe sepsis incidence, mortality, cost, and postsepsis syndrome. A summary of the result findings is highlighted in Table 1. 

## 3. Results 

### 3.1. Incidence of Sepsis

There were several reports that addressed the epidemiology of sepsis over the past three decades. Most trials continue to report on the increase in the incidence of sepsis [9,10,11,12]. Most recently, Stoller and colleagues examined 4-year epidemiological trends on severe sepsis utilizing data from the 2010 US Census [13]. Severe sepsis incidence increased annually (*p* < 0.05). The incidence of sepsis was remarkably higher among the oldest old (>85 years), with around a 30-fold higher incidence reported from a 10-year data evaluation of Taiwanese health insurance claims [12]. Additionally, Angus and colleagues conducted an observational cohort study and analyzed data retrieved from 850 nonfederal hospitals in seven US states [14]. There were 750,000 cases of sepsis per annum nationally, which is equivalent to a national incidence rate of 3 in 1000 individuals. The incidence rate per 1000 based on age ranged between 5/1000 for 60–64 years of age and 26/1000 for persons greater than 85 years of age. The results of this data analysis suggest that the overall number of cases of sepsis has increased faster than the anticipated population growth. Angus and colleagues commented on a 1.5% increase in the cases of sepsis per annum, which is equivalent to 900,000 in 2010 and 1.10 million in 2020 [14]. Recent data estimate around 1.5 million cases of sepsis in the United States. Dombrovskiy and colleagues conducted a trend analysis of severe sepsis data from 1993 to 2003 and commented on a rapid increase in the rate of hospitalization annually [15]. The percentage of cases of severe sepsis continuously increased throughout the years, ranging from 25% in 1993 to 44% in 2003 (*p* < 0.01). The age-adjusted rate of hospitalization with severe sepsis also steadily increased annually by 9% (*p* < 0.001). Wang and colleagues analyzed data from the National Hospital Ambulatory Medical Care Survey between 2001 and 2004 [16]. There were around 2.3 million cases of suspected severe sepsis, accounting for 570,000 annually. Martin and colleagues also commented on the annual increase in the incidence of sepsis [17]. This was a review of discharge data over a 22-year period which included data on 10 million cases of sepsis. There was an 8% annual increase in the incidence of sepsis, which accounted for 82/100,000 in 1979 and 240/100,000 in 2000 (160,000 cases vs. 660,000 cases). In subsequent data from the 2010 US Census that accounted for around 310 individuals and estimated the epidemiological trends between the years 2008 and 2012, the incidence of sepsis continued to increase from 346/100,000 to 436/100,000 (*p* < 0.05) [13]. Lastly, Gaieski and colleagues evaluated four nationally representative samples of data that were previously published on severe sepsis between 2004 and 2009 [18]. This study reinforces the reality surrounding the continuous increase of incidence of sepsis in the United States. There was an average increase in incidence of sepsis by 13% yearly. In summary, the incidence of sepsis significantly varies by age group and has been steadily increasing throughout the years. 

### 3.2. Hospital Length of Stay, Readmissions, and Mortality

Sepsis was one of the major risk factors identified that led to in-hospital death, discharge to hospice facilities, or 30-day readmissions [19]. Mortality related to sepsis was up to 140% higher compared to annual estimates of mortality from other causes [20]. Hall and colleagues reported on the results of their review of data from the National Hospital Discharge Survey, 2008 [21]. The average length of stay (LOS) for septic patients was 75% longer compared to those hospitalized for other conditions, and septic patients were eight times more likely to die during hospitalization. There were 17% in-hospital deaths among patients treated for septicemia compared to 2% of those treated for other conditions. This was similar to the 4-year epidemiological trends from the 2010 US census, which described a decrease in the overall mortality estimate from 22% to 17% over 4 years [13]. This was similar to the 2011 Nationwide Inpatient Sample, which estimated an all-cause mortality rate of around 15% and an average length of stay of 7 days [22]. A retrospective analysis of 12-year data on severe septic patients in Australia and New Zealand demonstrated a significant decrease in absolute mortality, estimated at 18% (*p* < 0.001) [2]. The account on readmission rates was also remarkable. Among Medicare patients, septicemia ranked second (after heart failure) as a condition with the highest 30-day readmission rates, accounting for 93,000 readmissions. These were substantial findings that have attracted the attention of stakeholders to devise strategies that aim at improving care coordination with the goal of decreasing the number of readmissions and achieving cost savings [23]. The in-hospital mortality data among septic patients remains remarkably high and worse mortality data are reported at 1 and 5 years posthospitalization with septic patients. The odds of death varied by age. In a retrospective analysis of data involving managed care organization enrollees between 1995 and 1999, the odds of death within the first year since hospital admission increased dramatically for the older group, reaching as high as 9 (*p* < 0.0001). Angus and colleagues reported on an overall hospital mortality rate of 28%, which is a number that is identical to septic ICU patients from the Finnsepsis study [14,24]. In this study, age was found to be an independent risk factor for sepsis mortality, where adults greater than 65 years had a two-fold increase in mortality compared to younger patients (40% vs. 20%). The 1-year mortality in this cohort was estimated around 40%, and mortality at 2 years was 1.5 times higher when compared to hospital mortality of 28%. The 1-, 2-, and 5-year mortality data continued to be remarkably high in several studies. In a retrospective study that examined the long-term mortality of severely septic patients, in-hospital mortality accounted for 21%, while 1-year hospital mortality doubled at 51%, and 5-year hospital mortality was estimated at 74% [25]. When comparing trauma and septic patients with similar Acute Physiology and Chronic Healthy Evaluation III (APACHEII) scores, in-hospital mortality was higher among ICU septic patients compared to trauma patients (58% vs. 38%, *p* = 0.002). Posthospital mortality of septic patients was also higher than trauma patients (22% vs. 8%, *p* = 0.049), as well as the 2-year mortality (67% vs. 43%, *p* = 0.002) [26]. When matched to a nonsepsis cohort, the 90-day and 1-year mortality was also significantly higher, accounting for 27% and 44% vs. 15% and 31%, respectively (*p* < 0.01). An observational cohort study that examined participants from the Health and Retirement Survey showed a remarkable use of healthcare resources after severe sepsis. The percentage of readmissions to the hospital at any point in time in the first year reached 63%. Also remarkable to note, patients spent a mean of 25% of their days alive in the first year following severe sepsis being admitted to an inpatient facility such as Long Term Acute Care (LTAC) or Skilled Nursing Facility (SNF) [27]. Finally, a more recent study conducted by Goodwin and colleagues highlighted the very high rate of readmissions among severe sepsis survivors [28]. In an observational cohort study that examined data from the Healthcare Cost and Utilization Project (HCUP), there were 26% and 48% readmissions after 30-day and 180-day postdischarge from severe sepsis respectively. The length of stay was comparable, with an average of 16–20 days [14,29]. 

### 3.3. The Cost Burden of Sepsis 

The cost of sepsis and postsepsis care continues to be a serious healthcare burden. Based on the 2013 HCUP statistical brief, sepsis costs accounted for $23 billion and was the most expensive condition treated in US hospitals [30]. A recent report evaluated by the CDC commented on the healthcare resources used, and it is estimated that 7 out of 10 patients who were once treated for sepsis will continue to utilize a variety of healthcare services or will have chronic illnesses that require frequent medical care. In 2011, sepsis was estimated to cost $20 billion annually or $55 million daily. This is considered the highest aggregate hospital cost and represents a quadrupled increase in cost or an increase of around 11% compared to data from 1997 [31]. The median hospital cost of sepsis was estimated at around $16,000 [22]. The cost of sepsis may also vary based on the etiology of sepsis (healthcare, community, or hospital sepsis), where the highest cost has been attributed to hospital-acquired severe sepsis. The cost of hospital-acquired severe sepsis was remarkably higher and estimated at $38,000 compared to the community-acquired cost of $7000 [32]. Sepsis care is a challenge to both patients and hospitals. A significant portion of healthcare costs stem not only from inpatient hospital costs but also from postsepsis care costs. The percentage of patients discharged to long-term care institutions is more than double when compared to patients hospitalized with conditions other than sepsis (36% vs. 14%) [31]. 

The increase in the cost of sepsis is indirectly related to the major increase in survivorship of severe sepsis. In a retrospective analysis of data pertaining to Medicare beneficiaries between 1996 and 2008, there was around a 120% increase in 3-year survivorship of severe sepsis, 16% of which had a moderate-to-severe cognitive impairment, and 75% or 500,000 patients had a functional disability. This is clearly an added cost that is indirectly related to sepsis survivorship. When compared to acute myocardial infarction (AMI), severe sepsis data are much more dramatic in regards to cost. While there was a small decrease in the cost of treatment over a decade for AMI patients, the cost of severe sepsis has more than doubled ($6 billion vs. $15 billion in 2008) [33]. These data are a clear representation of the population burden posthospitalization with severe sepsis and also aligns with other studies that demonstrated the threefold increase in the odds of developing cognitive impairment and the addition of functional limitations [33].

Comorbidities and acuity of illness played a substantial role in determining the cost of care for sepsis survivors. For example, the cost of care of patients with known diabetes and associated complications was more than double the cost of care of those without diabetes ($32,000 vs. $13,000). This is notably different when compared with the cost of care for patients with diabetes in the general population, which is estimated at $2300. When examined in terms of APACHE II score, patients with greater than or equal to a score of 25 incurred an average cost of $84,500. The cost of care also differed by the year postsepsis survival. The costliest care was accrued during the first year, which not only accounted for $14,000 but also reflected the cost of readmissions. This was much greater than the second and third year postsepsis, which accounted for $5000 [34]. 

As noted earlier, the cost of sepsis is not restricted to hospital care but is a reflection of the healthcare services received postdischarge as well as the cost of readmissions. In a retrospective analysis of data that involved managed care organization enrollees, the average cost of hospital care was $26,000 for a mean LOS of 16 days; however, these numbers differed drastically between medicine and surgery patients, with an estimated cost of $6000 and $35,000 respectively. In regards to posthospitalization costs, per patient per month (PPPM) outpatient and pharmacy costs were estimated to be around $1300 for sepsis survivors. Emergency room (ER) costs and inpatient visit costs certainly contributed to the overall costs, further complicating the healthcare burden imposed by sepsis survivors [29]. Data from US insurance claims between 1991 and 2000 also addressed the cost of severe sepsis, which was once again substantial. The admission cost of severe sepsis was estimated at $45,000, and the cumulative cost of care at 1 year and 5 years was estimated at $78,000 and $119,000, respectively. More recently, Goodwin and colleagues examined HCUP data and found that the average cost of readmission was $25,000 and the cumulative cost of readmissions amounted to $1.1 billion for those admissions at 180-days postdischarge, which accounted for 43,000 total admissions [28]. Finally, a record of hospitalizations between 2009 and 2011 were retrieved from HCUP and the State Inpatient Database (SID). This was a retrospective cohort analysis that examined the cost of 30-day readmissions. Sepsis was the costliest condition, with an estimated annual cost of $500 million, which is not nearly comparable to Congestive Heart Failure (CHF) and Acute Myocardial Infarction (AMI) costs of $229 million and $149 million, respectively. This discrepancy delineates the “centrality of sepsis” in regard to the readmission problem. 

Severe sepsis remains a burden on the US healthcare system and worldwide despite the available advances in technology and treatment [34,35,36,37,38,39,40]. This condition remains the costliest to treat, as its cost dramatically exceeds that of CHF and AMI. There is a notable difference between the cost of hospitalization of severe sepsis compared to all-cause admissions, and this reflects in many instances the burden imposed by the younger population on the healthcare system [29]. The overall cost of sepsis is not only a reflection of the hospitalization cost but also of other attributions such as readmissions and the chronic use of healthcare resources afterward. Additionally, survivorship of sepsis has played an important role in driving the increase in cost, as a great percentage of sepsis survivors have some sort of cognitive and functional disability. 

### 3.4. Postsepsis Syndrome

Postsepsis syndrome, as evidenced by cognitive and functional disabilities, is another major burden that is assumed by a great percentage of sepsis survivors. In a prospective cohort study that examined cognitive and physical functioning among severe septic patients as compared to nonsepsis hospitalized patients, there were significant differences found. Among severe sepsis survivors, the odds ratio of developing moderate-to-severe cognitive impairment was 3.3 as compared to no change in cognitive impairment among nonsepsis hospitalized patients (*p* = 0.01) [39]. Similarly, severe sepsis survivors developed more functional limitations compared to nonsepsis hospitalized patients (*p* = 0.001). The extent of limitations and the experienced decline in both cognitive and functional abilities persisted for 8 years postsepsis hospitalization [33]. In another study that evaluated around 800 critically ill patients, including septic shock patients, approximately 70% of patients had a decline in cognitive functioning and had global cognition scores that were similar to those patients with traumatic brain injury and mild Alzheimer’s disease condition [35]. This type of cognitive impairment is not only chronic but overburdening to patients and their families, and it is associated with high healthcare costs that are estimated in one study to be around $34,000 and $15,000 per year for patients and families, respectively [36]. Benros and colleagues (2015) evaluated around 160,000 men from the Danish Nationwide Register and concluded that men who were previously exposed to infection had a significantly lower cognitive ability (*p* < 0.001) [37]. This finding, while not addressing severe sepsis specifically, is highly relevant as it addresses the association of infection and change in immune system responses with significant decline in cognitive functioning. Finally, a systematic review of the literature that evaluated 12 studies also concluded that cognitive impairment is a major sequela that is observed among postsepsis survivors [38]. This is clearly a serious quality of life and cost burden issue that needs to be addressed, and measures to address prevention of cognitive decline should be established. This surely calls for several initiatives such as further research in the area of cognitive neurology, establishment of guidelines that address specifically the issue of postsepsis cognitive impairment (perhaps via instituting preventive management strategies in the early stages of sepsis), as well as further research that is much needed in regard to exploring variables or risk factors contributing to cognitive decline following critical illness. Considering the high cost of therapy of this overburdening condition, an increase in federal funding may be warranted. Such funding would involve the recruitment of specialists in the fields of occupational therapy and neurology as well as other scientists to pursue further research.

## 4. Conclusions

The burden of sepsis on our population has been reviewed and is clearly multifactorial. The incidence of sepsis has been steadily increasing over the past three decades and varies significantly by age group, with the most elderly patients carrying the greatest burden. Readmissions, length of stay, and mortality rates are all much higher in patients with sepsis when compared to patients admitted with nonsepsis diagnoses. This disparity in outcomes correlates to a substantially higher financial burden associated with this disease state. The cost of sepsis is both a reflection of the cost of initial hospitalization of these patients as well as care required postdischarge. Survivorship of sepsis has played an important role in driving the increase in cost, as a great percentage of sepsis survivors endure postsepsis syndrome and as such have cognitive and functional disabilities requiring significant healthcare resources long term. 

The impact on patient outcomes and the increasing incidence of sepsis is a serious quality-of-life and cost-burden issue that needs to be addressed. Measures to address prevention of cognitive decline have not been and should be established. Initiatives that may aid in the development of these preventative strategies include: further research in cognitive neurology, establishment of guidelines that address the issue of postsepsis cognitive impairment via institution of preventive management strategies in the early stages of sepsis, and further research exploring contributory variables associated with cognitive decline following critical illness. 

## Figures and Tables

**Table 1:** Review table of literature review findings.

Studies	Designs/Setting	Incidence			Mortality					Cost			Comments
		Not Specified	60–64	>65	In-Hospital	At 1 Year		At 2/5 Year		Hospital	Post Hospital	Readmissions	
Angus et al. (2001) [14]	Observational/50 nonfederal hospitals in US		5/1000	26/1000	Hospital mortality rate estimated at 28%					⚬ Surgical patients: 30K ⚬ Medical patients: 19 K (*p* < 0.0001)			1.5% increase in the cases of sepsis per annuum
Dombrovskiy et al. (2007) [15]	Trend analysis from 1993–2003												Percentage of cases of severe sepsis increased from 25% to 44%
Martin et al. (2003) [17]	Review of discharge data over 22 years and 10 million cases of sepsis	82/100,000 in 1979 vs. 240,000 in 2000											8% annual increase in the incidence of sepsis
Gaieski et al. (2013) [18]	Four national data between 2004–2009	13% yearly increase incidence of sepsis											
Hall et al. (2011) [21]	Review of 2008 National Hospital Discharge Survey				17% in-hospital deaths								Compared to 2% of deaths from conditions other than sepsis
Pfuntner et al., 2013 [31]	Data analysis of hospital costs in 2011									Highest aggregate cost of hospital among adults with septicemia estimated around $ 20 billion in 2011 or $ 55 million daily			This represents an 11% increase yearly since 1997
Wang et al. (2007) [16]	Analysis of data 2001–2004	⚬ 2.3 million cases of severe sepsis ⚬ 570,000 cases annually											
Lee et al. (2004) [34]	Analysis of data on 800 severe sepsis patients					12% death					Mean cost for year 1 was 14K–35K		⚬ Risk of death increased with age ⚬ PPPM outpatient and pharmacy cost was $ 1300
Weycker et al. (2003) [25]	Retrospective study. Data from US insurance claims 1991–2000				Estimate mortality: 21%	Doubled at 51%		Estimate mortality: 74%		Admission cost 45 K	⚬ At 1 year: 78K ⚬ At 5 year: 119K		⚬ 50% discharge home ⚬ 30% discharged to outside facility
Jagodic et al., (2006) [26]	Observational: long term survival of sepsis vs. trauma patients				⚬ Mortality 58 % vs. 38% (*p* = 0.002) ⚬ Post hospital mortality 22% vs. 8% (*p* = 0.049) ⚬ 2 years mortality 67% vs. 43% (*p* = 0.0002)								
Goodwin et al. (2015) [28]	Observational/data analysis/HCUP											⚬ Average cost: $ 25K ⚬ Cumulative cost at 180 days: $ 1.1 billions	⚬ 26% readmissions at 30 days ⚬ 48% readmissions at 180 days
Prescott et al. (2014) [27]	Observational 1998–2005 Health Retirement Survey							44% was the 1 year mortality					Significantly different than matched nonspesis cohort, 31% vs. 15% (*p* < 0.01)
Braun et al. (2004) [29]	Retrospective data analysis 1995–1999	⚬ <50 years old: 1 per 1000 ⚬ >50 years old: 4 per 1000			20% deaths. The odds of death were 9 for ages 80 and older					Average cost of $ 26K			
Karlsson et al. (2007) [24]	Prospective Study/24 ICUs and 21 hospitals						One year mortality: 40%		2 years mortality: 42%				2 fold increase in mortality for adults >65 years of age (40% vs. 20%)
